# Mediastinal Mature Teratoma Revealed by Empyema

**DOI:** 10.1155/2016/7869476

**Published:** 2016-04-07

**Authors:** Mohammed Raoufi, Laila Herrak, Anas Benali, Leila Achaachi, Mustapha El Ftouh, Salma Bellarbi, Charaf Tilfine, Firdaous Taouarsa

**Affiliations:** ^1^Avicenne University Hospital, Pulmonary Unit, International University of Rabat, Mfadel Charkaoui Street, BP 6527, Rabat, Morocco; ^2^Avicenne University Hospital, Anatomopathology Unit, International University of Rabat, Mfadel Charkaoui Street, BP 6527, Rabat, Morocco; ^3^Avicenne University Hospital, Radiology Unit, International University of Rabat, Mfadel Charkaoui Street, BP 6527, Rabat, Morocco

## Abstract

Teratomas are germ cell tumors, manifested with a great variety of clinical features; the most common extragonadal site is the anterior mediastinum. In this case, we report the patient with a large mature mediastinal teratoma with several components of ectodermal and endothermal epithelium. A 24-year-old female patient presented with history of persistent chest pain and progressively aggravating dyspnea for the previous 3 months. A chest X-ray showed a large opacity of the entire left hemithorax. Transcutaneous needle aspiration revealed a purulent fluid. The tube thoracostomy was introduced and the effusion was evacuated. Some weeks later, patient was seen in emergency for persistent cough and lateral chest pain. CT scan revealed a mass of the left hemithorax. The mass showed heterogeneous density, without compressing mediastinum great vessels and left hilar structures. Lipase value was elevated in needle aspiration. The patient underwent a total resection of the mediastinum mass via a left posterolateral thoracotomy. Microscopy revealed a mature teratoma with cystic structures. The patient subsequently made a full recovery. This case provide benign mediastinal teratoma with total atelectasis of left lung and elevated lipase value in needle transcutaneous aspiration; this event is explained by pancreatic component in the cystic tumor. Total removal of the tumor is adequate treatment for this type of teratoma and the prognosis is excellent.

## 1. Introduction

Germ cell tumors are pronominally found in gonads. Teratomas are classified to be composed of ectopic tissues from 2 or 3 germs layers, including mature, immature, or malignant components [1].

Most mediastinal teratomas produce no symptoms; they are more commonly associated with compression of mediastinal structures (great vessels, respiratory system).

Teratomas mostly occur in young adults, with an approximately equal incidence in males and females [2].

We present the case of a patient with mediastinal mature teratoma taken originally for empyema.

## 2. Patient and Observation

A 24-year-old female student patient was seen in the university hospital because of chest pain and dyspnea. The patient had been in her usual health until several months before presentation when dyspnea, dry cough, fatigue, and lateral chest pain developed.

The patient had no history of medical or surgical problems; she could independently perform activities of daily living, and she had no history of smoking or drinking alcohol.

On examination, the patient was normal, in no distress; she spoke with a normal voice.

The blood pressure was 120/70 mm Hg, the pulse was 64 beats per minute, oxygen saturation was 95% while she was breathing ambient air, the weight was 57 kg, and the body mass index was 20.2.

On chest auscultation, breath sounds on the left side were absent, with no clinically detectable lymphadenopathy; the remainder of the examination was normal.

**Figure 1:**
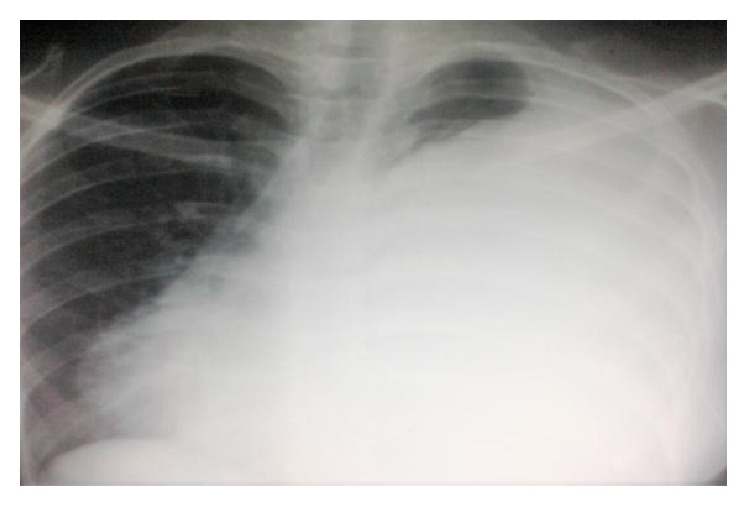
Chest X-ray shows a large opacity of the entire left hemithorax.

The chest X-ray showed a large opacity of the entire left hemithorax (Figure 1).

Thoracic ultrasound showed a large fluid collection of the left hemithorax.

**Figure 2:**
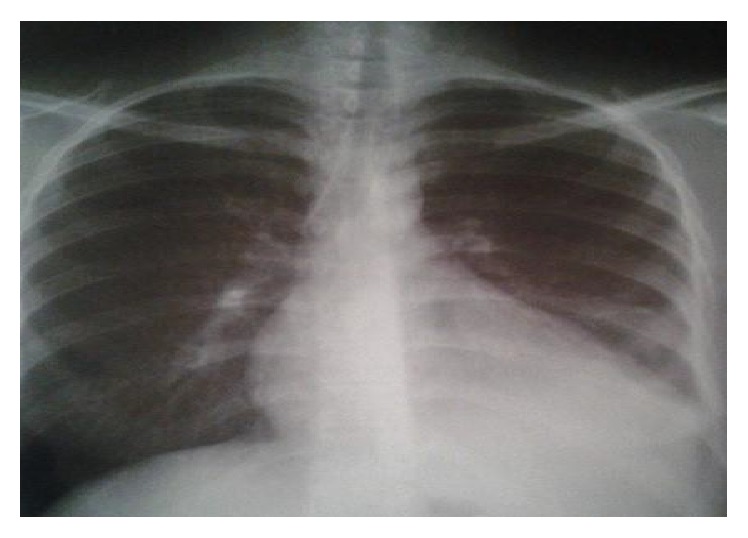
Control of chest X-ray after fluid evacuation.

In transcutaneous needle aspiration, the fluid removed was purulent. Therefore, tube thoracostomy was introduced, and the effusion was evacuated. The control of chest radiograph was good (Figure 2).

Cytological analysis shows the presence of many polymorphonuclear structures, some altered without suspect cell.

The Xpert MTB/RIF detects no DNA sequences specific for* Mycobacterium tuberculosis* in fluid aspiration.

Blood levels of glucose, total protein, creatinine, aspartate aminotransferase, and alanine aminotransferase were normal. Mantoux test, serology of HIV, and amebiasis were negative. Combination therapy with amoxicillin-clavulanic acid and ciprofloxacin was begun.

Bronchoscopy showed thickening of the interlobular spur and stenosis with extrinsic compression of the lingula's apertures. Bronchial biopsies revealed no specific inflammatory reshuffle.

Transthoracic echocardiography showed no pericardial effusion but heart was repressed by fluid chest training. ENT examination was normal. The patient had good recovery and was discharged home.

**Figure 3:**
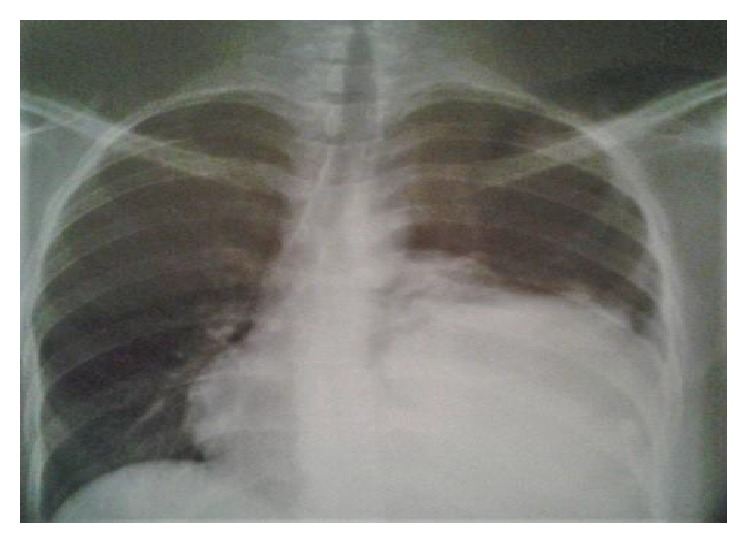
Chest X-ray shows opacity at the lower part of the left hemithorax.

Few weeks later, the symptoms worsened, and new dyspnea on exertion and cough developed. Chest X-ray revealed opacity at the lower part of the left hemithorax (Figure 3).

Biochemical analysis of fluid aspiration showed protein 51 g/L, glucose 2.5 mmol/L, amylase 14 U/L, lipase 4920 IU/L, hyaluronic acid 12.9 mg/L, and LDH 1820 U/L.

**Figure 4:**
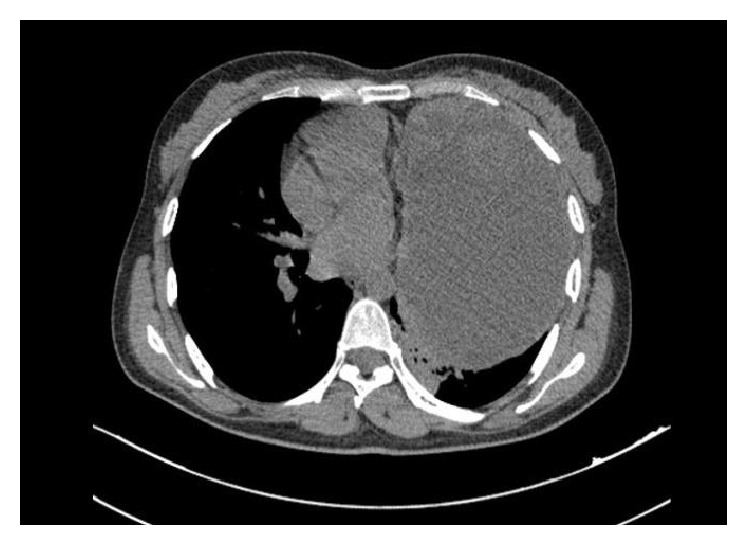
CT scan of chest shows a mass of the left hemithorax, which probably originated in the mediastinum and extended to the whole left pleural space.

CT scan was obtained and showed a mass of the left hemithorax, which probably originated in the mediastinum and extended to the whole left pleural space (Figure 4).

The mediastinum tumor markers (alpha fetoprotein and beta human chorionic gonadotropin) were both normal.

The patient underwent a total resection of the mediastinum mass via left posterolateral thoracostomy and entry into the pleural space was performed; the two pleural layers were intact.

Many adhesions existed within the left pulmonary artery, the pericardium, and the diaphragm.

Combination of blunt and sharp dissection for the division was applied uneventfully.

**Figure 5:**
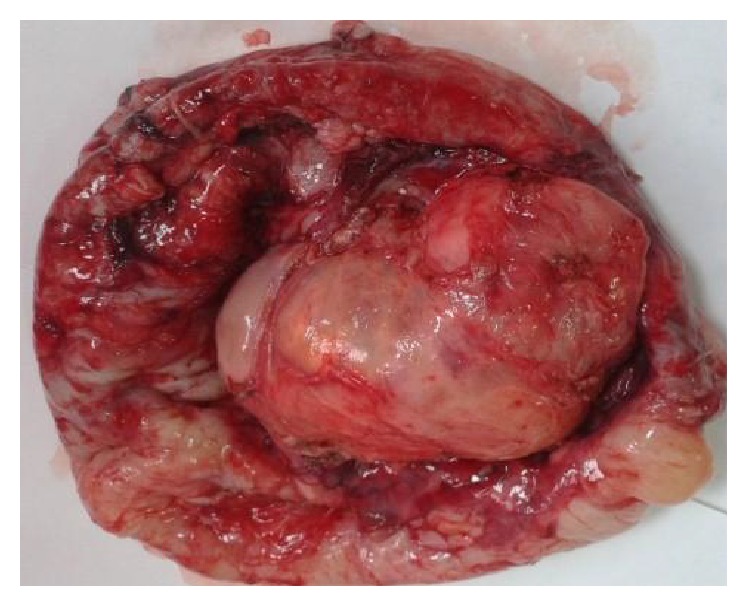
The tumor excised en bloc, pink-colored, and well circumscribed.

The tumor excised en bloc was pink-colored and well circumscribed (Figure 5).

A tube thoracostomy was introduced.

The collapsed left lung was easily reexpanded, the patient was extubated and recovered well from the operation, and she was discharged on the 6th postoperative day.

**Figure 6:**
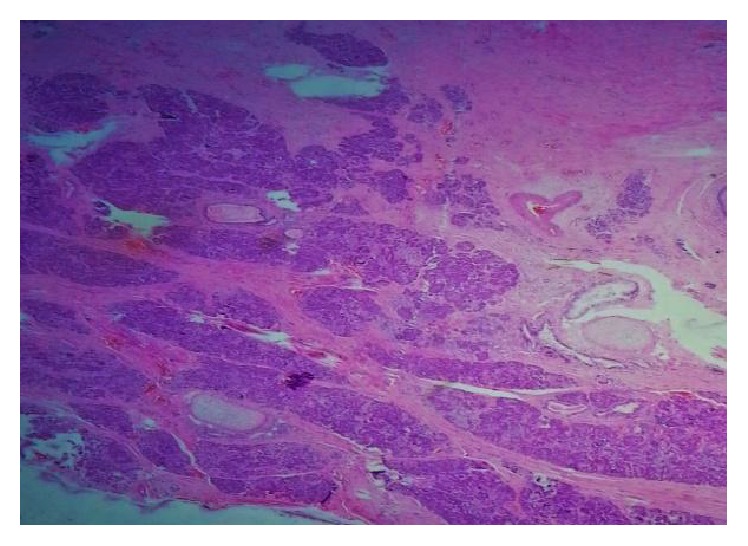
HE (hematoxylin and eosin) ×50 showed cartilaginous coating and pancreatic parenchyma.

**Figure 7:**
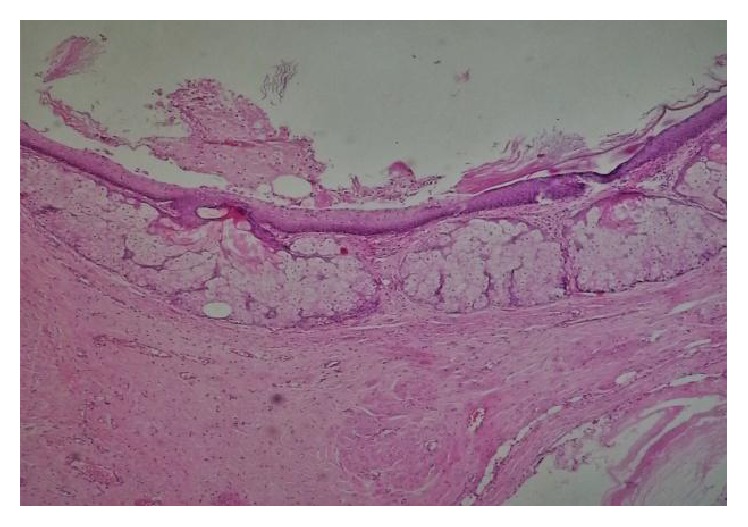
HE (hematoxylin and eosin) ×100 showed skin surface and sebaceous glands.

Pathological examination of the mass revealed mature teratoma with cystic structures lined by stratified squamous epithelium and at places cartilage, respiratory epithelium, pancreatic epithelium, urothelial epithelium, mucous secreting glands, muscle fibers, and epidermal cyst (Figures 6 and 7).

## 3. Discussion

Teratomas are germ cell tumors and they are uncommon neoplasms that arise in the gonads; the mediastinum is the second most common extragonadal site of these tumors [3, 4].

They usually occur in the middle of the body, which is the route of germ cell migration during embryogenesis; their migration can be misplaced en route to their appropriate organs, leading to the development of tumors later in life [5].

In fact, several theories exist; one of them suggests that benign teratoma is derived from the region of the third bronchial cleft or pouch. A second theory states that these tumors arise from germinal nests of cells located along the urogenital ridge that failed to migrate to the gonads in embryological development [6].

Primary mediastinal nonseminomatous germ cell tumors (NSGCT) are uncommon neoplasms and are clinically and biologically distinct from other germ cell tumors, and they are classified as a poor prognosis group in the IGCCC [7].

Rivera et al. demonstrate two prognosis factors of those tumors: the presence of residual viable cancer in tumor residue and the positivity of serum tumor markers (*α*FP or *β*HCG) after surgical resection [8]. The multimodality approach to primary mediastinal nonseminomatous germ cell tumor with intensive cisplatin-based chemotherapy, emphasis on normalizing serum tumor markers, and aggressive resection of residual disease offers survival to a significant number of patients [9].

Benign teratomas are often asymptomatic and are discovered on chest radiograph for unrelated reasons, but sometimes they give symptoms leading the patient to consult. In the case reported, the patient had chest pain, dyspnea, and dry cough.

If symptoms are present, this will be because of the mass effect caused by mediastinal teratoma.

Rarely, a patient may have expectoration of hair, and this is a pathognomonic symptom. However, this distinctive symptom is extremely rare and occurs late in the natural history of this condition following tumor rupture into the tracheobronchial tree. Laboratory exams are often normal, and serum levels of human chorionic gonadotropin and alpha-fetoprotein are always normal in patients with benign teratoma.

Radiology exams reveal usually a well-circumscribed mediastinal mass that often protrudes into one of the lung fields.

Pleural effusion also can be the most common ancillary CT finding in ruptured mediastinal teratomas. Pericardial effusion, especially in patients with a teratoma adhering to the pericardium, also appeared to suggest rupture of the tumor into the pericardium.

In the case under discussion, CT scan showed a mass of the left hemithorax extended to the whole left pleural space.

High level of amylase or lipase can be noted in pleural fluid that originated from a mediastinal teratoma and suggest pancreatic component in mediastinal mass.

Surgical excision is the treatment of choice for mediastinal teratomas.

The aim of surgery is to achieve complete clearance of the tumor without damage to associated or adjacent structures. This requires a careful and meticulous surgical dissection.

The prognosis of mature teratomas of the mediastinum is excellent after complete resection. Recurrences are rare, especially related to incomplete resection [10–12].

## Abbreviations

MTB/RIF: * Mycobacterium tuberculosis*
DNA: Deoxyribonucleic acid.
